# Large-scale spatial variation in feather corticosterone in invasive house sparrows (*Passer domesticus*) in Mexico is related to climate

**DOI:** 10.1002/ece3.1638

**Published:** 2015-08-21

**Authors:** Gillian D Treen, Keith A Hobson, Tracy A Marchant, Gary R Bortolotti

**Affiliations:** 1 Department of Biology, University of Saskatchewan112 Science Place, Saskatoon, SK, Canada, S7N 5E2; 2 Environment Canada11 Innovation Boulevard, Saskatoon, SK, Canada, S7N 3H5

**Keywords:** Climate, corticosterone, ecophysiology, feather CORT, invasive species, macrophysiology, Mexico, *Passer domesticus*, precipitation, temperature

## Abstract

Ecologists frequently use physiological tools to understand how organisms cope with their surroundings but rarely at macroecological scales. This study describes spatial variation in corticosterone (CORT) levels in feathers of invasive house sparrows (*Passer domesticus*) across their range in Mexico and evaluates CORT–climate relationships with a focus on temperature and precipitation. Samples were collected from 49 sites across Mexico. Feather CORT (CORT_f_) was measured using methanol-based extraction and radioimmunoassay. Relationships between CORT_f_ and spatial and climate variables were examined using simple linear regressions. Ordination was used on climate data, CORT_f_ was plotted against the resulting axes, and univariate regression trees were used to identify important predictors of CORT_f_. Universal kriging interpolation was used to illustrate spatial variation in CORT_f_ across Mexico. Correlations with ordination axes showed that high CORT_f_ was associated with low precipitation during the rainy season and low dry season temperatures. Specifically, CORT_f_ was negatively related to May precipitation and January and July minimum temperatures, and positively related to April deuterium excess and June minimum temperatures. CORT_f_ was higher in second-year birds compared to after-hatch years and after-second years. House sparrows had higher CORT_f_ levels in the hot, dry, north-central region of Mexico, and CORT_f_ was negatively related to temperature and precipitation. House sparrows molt primarily from August–September but climate conditions throughout the year were important predictors of CORT_f_, suggesting that conditions outside of molt can carry over to influence energetics during feather growth. These data suggest that dry conditions are challenging for house sparrows in Mexico, supporting previous work showing that precipitation is an important predictor of broad-scale CORT variation. This work highlights the utility of CORT_f_ for evaluating the influence of physiology on current avian range limits; furthermore, these data may allow us to predict future changes in species distributions.

## Introduction

Combining ecology and physiology has led to considerable advancement in these fields, and the advantages of using ecophysiological data collected at broad scales to answer biological questions have long been recognized (Chown et al. 2004). Macrophysiology emphasizes variation in physiological traits at large scales, information which can be used to generate novel, holistic answers to basic questions in ecophysiology. For example, assessing current patterns can help us understand species distribution limits and predict future responses to climate or land-use changes (Chown and Gaston 2008). This approach is especially powerful for invasive species, as understanding what factors limit their range expansion is key to planning where to target management and what types of interventions will be most effective. It may also help determine how current species' ranges could expand or shift in response to predicted climate variation (Zuckerberg et al. 2011).

By linking broad-scale variation in physiological variables to climate, we can infer how well organisms are coping with their environments. The glucocorticoid (GC) hormone axis in particular is likely to be a physiological process that reflects the effects of climate. GCs are involved in energy regulation, especially processes that provide energy for routine daily tasks such as glucose synthesis and fat breakdown (Dallman et al. 1993; Toates 1995). Corticosterone (CORT) is the main avian GC and is often incorporated into ecological studies of birds because it is secreted in higher amounts when an animal experiences an unpredictable environmental perturbation or stressor (Wingfield et al. 1998; Romero 2004). Previous work has shown that plasma CORT (CORT_p_) levels are higher following storms or periods of extreme precipitation (Rogers et al. 1993; Smith et al. 1994; Astheimer et al. 1995; Boyle et al. 2010), and CORT_p_ and fecal CORT increases have also been associated with cool temperatures (Frigerio et al. 2004; Jenni-Eiermann et al. 2008; Lobato et al. 2008), cool, rainy conditions (Bize et al. 2010), and prolonged bouts of precipitation (Pereyra and Wingfield 2003). Furthermore, song wrens (*Cyphorhinus phaeocephalus*) had higher CORT_p_ levels in dry areas near their range limits on the isthmus of Panama, suggesting that they are unable to cope easily with these conditions and that this limited further expansion (Busch et al. 2011).

CORT can be measured in plasma, feces, or feathers, but there are several advantages to using feather CORT (CORT_f_). Blood measures provide an instantaneous picture of individual hormone levels, whereas CORT_f_ offers a longer-term perspective, integrating both baseline levels and any elevations occurring during the period of feather growth (Bortolotti et al. 2008). CORT_f_ also allows investigators to avoid the potential negative effects of blood sampling on survival (Brown and Brown 2009) and the difficulty of obtaining baseline blood samples in the field (Romero and Romero 2002; Romero and Reed 2005). CORT–climate relationships can vary with species, life-history stage, sex, age class, and social status. For example, Wingfield (1985a,b) found that an early spring storm was associated with increased CORT_p_ in female but not male song sparrows (*Melospiza melodia*), while during a late spring storm, males but not females showed elevated CORT_p_ levels. Rubenstein (2007) found that rainfall during the pre-breeding period was negatively related to CORT_p_ in subordinate superb starlings (*Lamprotornis superbus*), while dominant individuals showed no relationship (for other examples, see Schwabl et al. 1985; Rohwer and Wingfield 1981; Romero et al. 2000). Furthermore, CORT_f_ levels of common eiders (*Somateria mollissima*) in Nunavut, Canada, were positively related to August and September temperatures, and these variables explained the majority of the interannual variation in CORT_f_ (Legagneux et al. 2013). CORT_f_ has also been correlated with clutch size, social signals, and habitat conditions (Bortolotti et al. 2008; Harms et al. 2010; Fairhurst 2011) and associated with future survival probability of wild house sparrows (*Passer domesticus*) on an island off the coast of England, suggesting that it may be a useful biomarker for conservation (Koren et al. 2012).

Here, we present the first exploration of CORT_f_ variation in a single species at a broad spatial scale. Our primary aim was to look for patterns in house sparrow CORT_f_ across their range in Mexico and identify potential drivers of this variation. Specifically, we assessed relationships between CORT_f_ and spatial and local climate variables operating over various time scales. We predicted that CORT_f_ levels would be highest in birds living in low precipitation areas, similar to the findings of Busch et al. (2011). Also, we expected to see negative relationships between CORT_f_ and both elevation and latitude. We predicted that CORT_f_ would be negatively related to temperatures and precipitation levels during the molt period, defined as August–September (Casto 1974; Lowther and Cink 2006). This relationship with temperature could reflect direct effects of cool temperatures on energetic demands or indirect effects of temperature on food availability. Associations between rainfall and CORT_f_ are likely indirect, mediated through the effects of rainfall on food supply, as previously hypothesized in CORT_p_ studies (e.g., Bize et al. 2010; Busch et al. 2011).

## Methods

### Study species and study area

House sparrows are native to Europe and Asia but were introduced to the United States in the 1850s, and their New World range now extends from northern Saskatchewan and Manitoba, Canada, to Panama (Lowther and Cink 2006). House sparrows spread across the majority of Mexico between 1910 and the 1970s (Robbins 1973; Schrey et al. 2011). Their success as an invasive species has been partly attributed to the fact that they are generalist feeders and human commensals (Lowther and Cink 2006). The human population of Mexico has increased rapidly since the 1940s, and the percentage of the population living in urban areas increased from 35.1% in 1940 to 78% in 2010 (United Nations 2012). Although most house sparrows in Mexico likely reside in urban areas or near rural dwellings, climate and physical geography vary considerably across the country. The coasts and the Yucatan peninsula are characterized by warmer temperatures (mean annual temperature >22°C), while the majority of the interior is cooler (mean annual temperatures ranging from 12 to 22°C; in some high-altitude regions, mean annual temperatures range from <5 to 12°C; Alemán and García 1974; Rudolph 1985). The Sonoran and Chihuahuan deserts are located in north-central Mexico, and along with the Baja peninsula, these regions are arid and subject to extreme high temperatures (mean July temperatures can range from 25 to 30°C) and considerable annual temperature variability (range of 16–20°C; Alemán and García 1974). Mean annual rainfall is highest on the Yucatan peninsula (range of 116–131 cm) and in the southern and central regions of Mexico (range of approx. 38–115 cm), while the northern plateau region and the Baja peninsula are more arid (mean annual levels 11–27 and 1–10 cm, respectively; Alemán and García 1974; Rudolph 1985).

### Field methods and climate data

Feathers were collected from December 2006 to March 2007 as part of a study that developed a feather *δ*^2^H isoscape for Mexico (Hobson et al. 2009). Sampling sites (*n* = 49) were chosen based on obtaining adequate coverage of the country and on accessibility from roadways. Birds were captured using mist nets, and individuals were sexed and assigned an age class (hatch year, HY; after-hatch year, AHY; second year, SY; after-second year, ASY; or unknown, U). Unflattened wing length was measured to the nearest mm. The number of individuals sampled per site ranged from 1 to 20, with a mean and mode of 9. Feathers were stored in paper envelopes at room temperature until they were used for hormone analysis in 2011.

Monthly precipitation, mean annual precipitation, and average monthly minimum and maximum temperatures for each sampling site were obtained from high resolution (1-km spatial resolution) interpolated global climate surfaces developed by Hijmans et al. (2005). Monthly deuterium excess in groundwater (*d*-excess = *δ*^2^H – 8*δ^18^O; Clark and Fritz 1997) was calculated for each site based on water samples as described in Wassenaar et al. (2009). Deuterium excess can be used as a proxy for evaporative conditions (Clark and Fritz 1997); we included this variable in addition to precipitation data because *d*-excess may be more representative of how much water is actually available in the local food web. It is likely that water availability rather than precipitation levels strongly affect factors influencing CORT secretion, such as dehydration or food availability.

### Corticosterone analysis

For each individual, the first secondary (S1) was typically sampled (*n* = 438), but the outer rectrix was used in 10 cases. CORT levels did not differ significantly based on which feather was used (Welch two-sample *t*-test, *P* = 0.22). Prior to analysis, the calamus was removed and each feather was measured to the nearest mm using a ruler. CORT_f_ values are reported as pg/mm, based on evidence that CORT is deposited into feathers in a time-dependent rather than a mass-dependent fashion (Bortolotti et al. 2008; Bortolotti 2010). After measurement, feathers were cut into small pieces (<5 mm^2^) using scissors. CORT was recovered from feathers using a methanol-based extraction technique described and validated in Bortolotti et al. (2008). To evaluate the efficiency of the recovery procedure, three feather samples spiked with ∼5000 CPM of ^3^H-corticosterone (Perkin Elmer, Woodbridge, ON) were included in each recovery. The 448 feather samples were recovered in 5 batches; for all batches, >90% of the radioactivity was recoverable from the reconstituted samples (mean recovery efficiency 95.8%, SE ±1.28%). Final CORT_f_ values were adjusted to account for recovery efficiency. Samples were stored in a −20°C freezer prior to radioimmunoassay.

Reconstituted samples were analyzed via standard radioimmunoassay procedures (Wayland et al. 2002), with each sample analyzed in duplicate. To avoid bias, samples were placed in random order prior to analysis and the investigator was blind to the site at which each sample was collected. Serial dilutions of feather extracts were shown to parallel the CORT standard curve (see Appendix S2 in Supporting Information), indicating that there were no substances in the extracts that compromised the assay (Buchanan and Goldsmith 2004; Bortolotti et al. 2008). To evaluate assay variability, three internal standards of known hormone concentration were included in each assay. Samples were processed in 10 assays, with a mean intra-assay coefficient of variation (CV) of 6.57% (range 4.50–10.6%) and an interassay CV of 4.63%. The mean detection limit (80% bound) was 10.0 pg CORT per 100 *μ*L of sample, and all data values exceeded this limit (mean = 43.0 pg/100 *μ*L, range 17.2–237).

### Statistical analyses

Data exploration was conducted following Zuur et al. (2010), including outlier analysis and evaluation of heteroscedasticity and collinearity of explanatory variables. The distribution of CORT_f_ in the sample was plotted; other preliminary analyses included using *t*-tests and ANOVAs to explore relationships between CORT_f_ and sex and age class, and using simple linear regressions, controlling for sex, to evaluate relationships between CORT_f_ and wing length.

To explore spatial variation in CORT_f_, correlations between CORT_f_ and latitude, longitude, and elevation were evaluated using simple linear regressions. *T*-tests were used to evaluate variation in CORT_f_ between the Atlantic and Pacific drainage basins and between the interior and exterior regions of Mexico (see Hobson et al. 2009). We tested for differences in CORT_f_ related to evapotranspiration using ANOVA; simple linear regressions were used to test for one-way relationships between CORT_f_ and deuterium excess or monthly climate variables. We applied a Bonferroni correction to account for multiple testing, with *P* < 0.0008 considered significant. Universal kriging interpolation was used to illustrate spatial variation in CORT_f_ across Mexico.

We used ordination to reduce the dimensionality of the climate data. Climate data were non-normal and nonlinear so we used non-metric multidimensional scaling (NMDS; McCune and Grace 2002). The Euclidean distance metric was used as it minimized the loss function compared to other metrics considered, and a final solution with two dimensions (axes) was chosen based on the same criterion (minimal loss function). CORT_f_ was plotted against the ordination axes, and correlations between original variables and axis scores (similar to factor loadings produced by PCA) were used to interpret the climate conditions represented by each axis.

Univariate regression trees were used to identify the most important predictors of CORT_f_. Trees were “pruned” by plotting tree size and then selecting the tree with the lowest error value. Each tree was run 10 times, and each run included 1000 cross-validations. Cross validation error values estimate prediction error of the model, and error averaged over all runs is reported for each tree. All statistical analyses were performed in R v. 2.14.0 and the “mvpart” library was used for regression trees (R Development Core Team 2012); kriging interpolation was carried out in ArcMap v. 10.1.

## Results

CORT_f_ levels did not differ significantly between the sexes (males 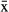 = 5.8 ± 2.9 pg/mm, *n* = 257; females 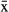 = 5.6 ± 2.1 pg/mm, *n* = 191; *P* = 0.42) or age classes (ANOVA, df = 4, *P* = 0.74). CORT_f_ levels were not strongly related to wing length for either sex (males *R*^2^ = 0.007705, *F* = 1.833, df = 236, *P* = 0.1771; females *R*^2^ = 0.0003422, *F* = 0.06229, df = 182, *P* = 0.803).

**Figure 1:**
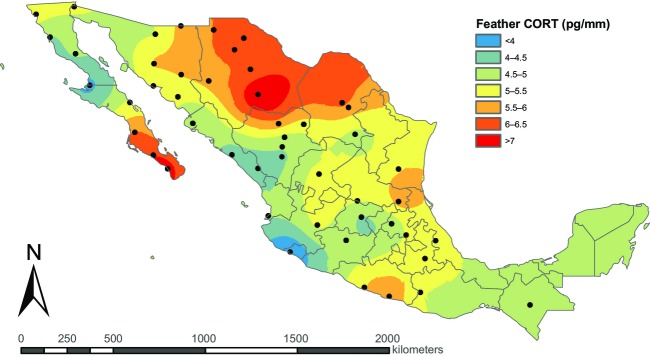
Kriged surface of feather corticosterone (CORT_f_) values of house sparrows (*n* = 425) sampled from 49 sites in Mexico. Universal kriging was used and data are shown with a cylindrical equal-area map projection. Birds with CORT_f_ levels above the 95th percentile (ranging from 9.6 – 32.4 pg/mm; *n* = 23) were considered “outliers” and were removed from the data set prior to analysis. CORT_f_ values were averaged for each site (sample size varied between sites; minimum *n* = 1, maximum *n* = 19, median *n* = 8).

The majority of the individuals sampled had CORT_f_ levels ranging from 2.5 to 10 pg/mm. Individuals with CORT_f_ levels above the 95th percentile (*n* = 23) were considered “outliers,” and their CORT_f_ levels ranged from 9.6 to 32.4 pg/mm. These individuals did not consistently belong to one sex or age class. However, they were all sampled at one of seven sites, with 12 captured at a single site. Additionally, 22 of the 23 individuals with high CORT_f_ values were sampled in the interior of Mexico and in the Atlantic drainage basin. CORT_f_ values were highest in north-central Mexico and the Baja peninsula and slightly lower in central Mexico compared to the rest of the country (Fig.1).

**Figure 2:**
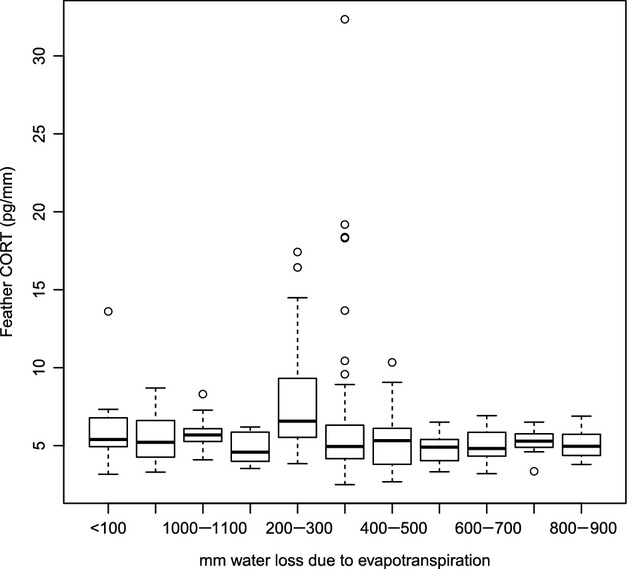
The relationship between feather corticosterone (CORT_f_) and evapotranspiration level in house sparrows (*n* = 425) sampled form 49 sites across Mexico; CORT_f_ levels differed significantly based on evapotranspiration level (ANOVA, df = 11, *F* = 4.46, *P* < 0.0001).

One-way associations between CORT_f_ and latitude, longitude, and elevation were weak (*R*^2^ values all <0.045) although the regression with latitude was significant (*P* < 0.0001). CORT_f_ values differed significantly between east and west drainage basins (Atlantic 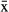 = 6.4 ± 3.3 pg/mm, *n* = 213; Pacific 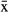 = 5.1 ± 1.4 pg/mm, *n* = 235; *P* < 0.0001) and between the interior and exterior drainage basins (as described in Wassenaar et al. 2009; interior 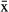 = 6.4 ± 3.5 pg/mm, *n* = 192; exterior 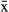 = 5.2 ± 1.3 pg/mm, *n* = 256; *P* < 0.0001). After removing 23 outliers from the data set, the difference was not significant for either comparison (Atlantic versus Pacific *P* = 0.003; interior versus exterior *P* = 0.071). CORT_f_ also differed significantly based on evapotranspiration level (ANOVA, df = 11, *F* = 4.46, *P* < 0.0001), with higher mean CORT_f_ values associated with low evapotranspiration values (<100 and 200–300 mm water loss due to evapotranspiration, Fig.2). Regressions modeling relationships between CORT_f_ and monthly precipitation, minimum and maximum temperature, and *d*-excess values did not reveal strong one-way associations (all *R*^2^ values <0.08); however, several models had significant *P*-values at the *α *= 0.0008 level (see Appendix S1).

The first ordination axis (NMDS1) was positively associated with precipitation during the molt period (in house sparrows molt primarily occurs in August and September, although more conservative estimates define the molt period as June–November; see Casto 1974; Mathew and Naik 1986; Lowther and Cink 2006; Romero et al. 2006) which overlaps with the rainy season in Mexico (July–September). NMDS1 was also positively associated with minimum and maximum temperatures of months outside of the molt period, in the dry season. The second axis (NMDS2) was negatively associated with precipitation during the molt period/rainy season and positively associated with minimum and maximum monthly temperatures during the molt period/rainy season. High CORT_f_ values were associated with low values of NMDS1, which corresponds to dry conditions during the molt period/rainy season and cool temperatures during the dry season (see Appendix S1). Mid-range values of NMDS2, which correspond to intermediate temperatures and levels of precipitation during the molt period/rainy season, were also associated with high CORT_f_. Further examination showed that some high CORT_f_ values were also associated with high values of NMDS2, which correspond to low levels of precipitation during molt, and mid-range values of NMDS1, which correspond to intermediate levels of precipitation during the molt period.

**Figure 3:**
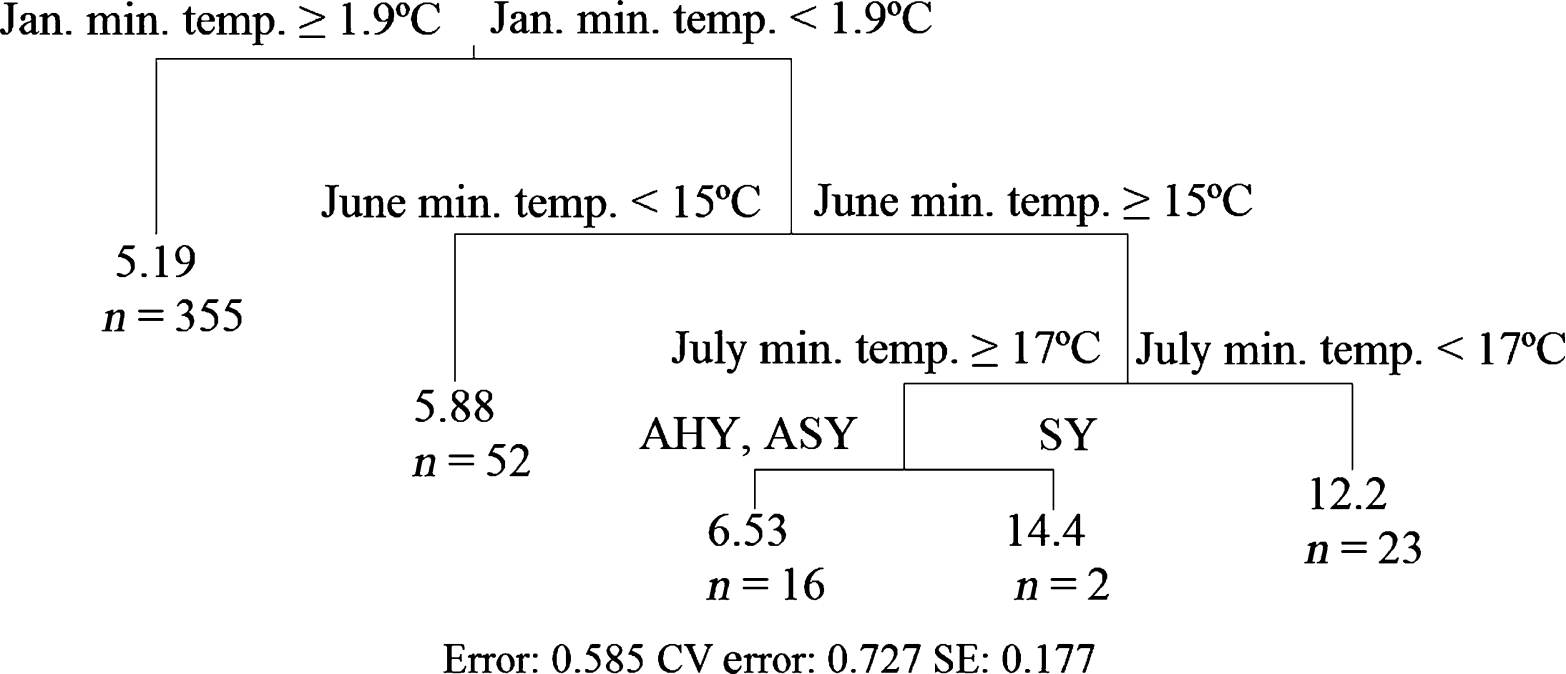
Regression tree showing the division of 448 house sparrows (*Passer domesticus*) sampled across Mexico into groups based on feather corticosterone (CORT_f_), with group divisions determined based on one of 45 predictor variables, each describing spatial location or climatic conditions of sampling sites. For each terminal node, the group size (*n*) and the mean CORT_f_ for that group (in pg/mm) are listed. Threshold values for each split are also listed; for temperature variables, units are °C.

**Figure 4:**
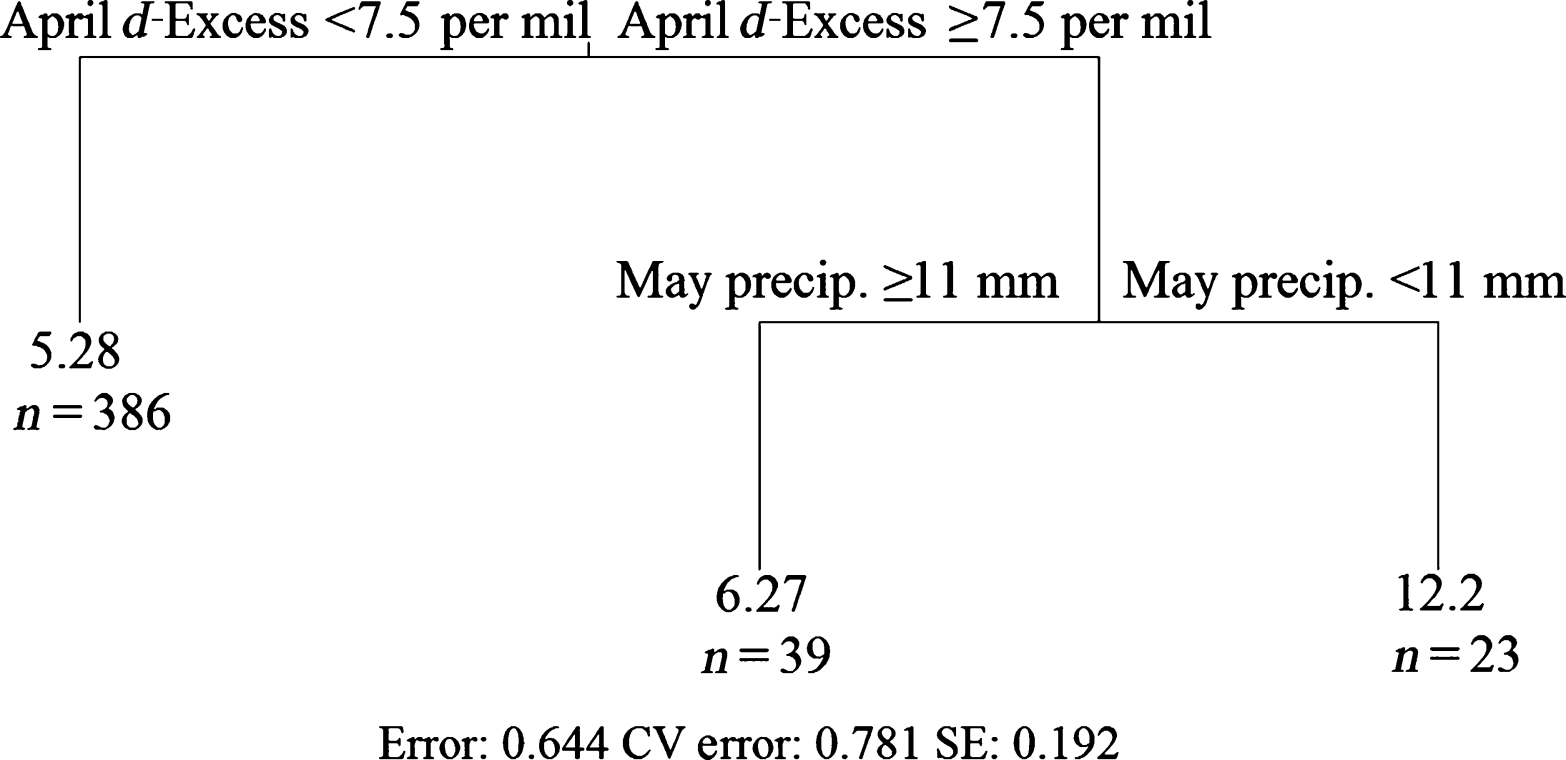
Results of rerunning the regression tree analysis shown in Figure3 with monthly deuterium excess included as predictor variables.

Regression trees were run with and without site groundwater *d*-excess data. In all cases, the most important variable predicting CORT_f_ was site. To determine whether the dominance of site was driven by seven sites from which outliers were sampled, a stepwise elimination of these sites was conducted with regression trees run after each removal. After removing these sites, site was still the top predictor in all trees. Age, mean annual precipitation, and *d*-excess also emerged as important predictors of CORT_f_. For the data set excluding *d*-excess, the modal best tree size from the 10 runs considered was three nodes, and both splits were based on site. Regression trees run without site identified minimum temperatures in January, June, and July, and age class as important predictors of CORT_f_ (Fig.3). Rerunning regression tree analyses of the data set including *d*-excess data after removing site identified *d*-excess in April and May precipitation as important predictors of CORT_f_ (Fig.4). As some of the climate variables were collinear, we also looked at surrogate variables for each split for the trees developed after removing site. Generally, the surrogate variables were quite similar to the variable on which the split was based. However, in some cases, precipitation variables or spatial data (latitude or longitude) appeared as surrogates for temperature variables, or vice versa.

## Discussion

House sparrows in Mexico had higher CORT_f_ levels in areas characterized by low precipitation and increased temperature variability. Overall CORT_f_ was negatively related to both temperature and precipitation, and weather conditions throughout the annual cycle were important predictors of CORT_f_. As CORT_f_ can be interpreted as a measure of energetic demand, our results indicate that weather may be an important factor limiting the distribution of this invasive species. More broadly, this work shows that linking CORT_f_ to environmental variables can identify conditions that are difficult for a species to cope with, which is likely an important determinant of the boundaries of the species' current range. Expanding upon this work, if CORT_f_ can be linked to survival, it may prove useful for predicting the extent of future range shifts or expansions.

To our knowledge, this study is the first to establish how intraspecific CORT levels vary across a broad geographical range and adds to a growing body of evidence confirming that CORT_p_–climate relationships can be replicated using CORT_f_ (see Fairhurst et al. 2012, 2013; Legagneux et al. 2013). While variation in house sparrow CORT_f_ across their range in Mexico was generally subtle, small differences in CORT levels can have fitness consequences (Romero and Wikelski 2001; Fairhurst 2011; Koren et al. 2012). Individuals with extremely low or extremely high CORT_p_ values are compromised in their ability to cope with their environment and often do not survive (Romero et al. 2009), reducing the probability of observing greater ranges of variability in CORT_f_. Alternately, because house sparrows tend to live in close associations with humans (see below), they may be “buffered” from environmental variation and as a result show fairly consistent CORT_f_ levels, rather than CORT_f_ levels seeming consistent due to reduced survival of individuals at either extreme.

Many studies have shown that sex, age, and body condition can influence plasma and fecal CORT (e.g., Bonier et al. 2007; Cabezas et al. 2007; Wilcoxen et al. 2011), but CORT_f_ did not differ significantly between the sexes or age groups and was not significantly associated with wing length in our study. One explanation for the uniformity of CORT_f_ levels of birds sampled across the country could be that house sparrows are human commensals. As such, their habitats may be fairly consistent in terms of food supply, shelter, predation pressure, and other factors affecting energetic requirements. The north-central region of the country and to an extent the Baja peninsula appear to be exceptions to this hypothesis, perhaps suggesting that in these areas, birds are less buffered by associations with humans. Studies have found that other urban bird species show CORT_p_ differences based on sex (Bonier et al. 2007), age class (De Neve et al. 2010), and body condition (Fokidis et al. 2011) despite their close association with humans. A primary aim of this study was to create a large-scale “feather CORTscape,” which required sampling a species with a broad distribution. However, it would be interesting to conduct a similar study on a species that is less associated with humans, to determine whether their CORT_f_ levels are more strongly linked to environmental variables.

The dominant spatial pattern in CORT_f_ was the clustering of the high CORT birds in the north-central region of Mexico, where temperatures vary considerably over the course of the year relative to other parts of the country (mean annual temperatures ranging from 12 to 22°C) and conditions are dry (mean annual rainfall ranging from 11 to 27 cm; Alemán and García 1974). Birds with high CORT_f_ levels were also sampled near the tip of the Baja peninsula, where conditions are similar, dry with variable temperatures. The tendency for CORT_f_ levels to be higher in the driest parts of a species' range is similar to previous findings using CORT_p_ of song wrens (Busch et al. 2011), and a positive CORT_f_–temperature relationship has been shown in common eiders (Legagneux et al. 2013). The pattern we observed in the present study could result from the combined challenges of hot and dry conditions in this area. Alternatively, high temperatures alone could be perceived as a stressor or increase energetic demands due to thermoregulation. While our results suggest a potential physiological mechanism linking weather conditions to house sparrow range limits in Mexico, specific data on weather conditions at the species' range limits are necessary to further investigate this connection.

Contrary to our a priori predictions, CORT_f_ showed a weak, positive relationship with latitude. Other work has shown no effect of latitude on CORT_p_ (Lynn et al. 2003), CORT_p_–latitude relationships that varied between years (Lindström et al. 2005) and positive relationships between latitude and CORT_p_ (Wingfield et al. 1995; Martin et al. 2005), suggesting that the latitudinal effect on CORT is variable. As birds sampled in desert-like northern Mexico had higher CORT_f_ levels, the positive CORT_f_–latitude relationship may reflect a negative association between latitude and rainfall in the study area. Similarly, higher latitudes could be associated with increased temperature variation and less stable temperatures with increased CORT_f_. Our study found an inconsistent association between elevation and CORT_f_ in house sparrows, with the intermediate elevations associated with the highest CORT_f_ levels. Previous studies have found a negative relationship between CORT_p_ and elevation (Bears et al. 2003; Pereyra and Wingfield 2003; Li et al. 2008). It is difficult to conceive of a biological explanation for our results, and it is possible that there are site specific variables that we did not capture in the present analysis. Alternatively, house sparrows may be somewhat “buffered” from altitude effects on the GC axis because they often live in close association with humans, and therefore, the availability of food, protection from adverse weather conditions, etc. may be similar across their altitudinal range.

CORT_f_ levels were significantly higher in the Atlantic versus the Pacific drainage basin and in the interior versus the coastal region. The majority of the high CORT_f_ outliers were sampled in the interior/Atlantic drainage basin; after removing these outliers from the data set, the Atlantic versus Pacific comparison still yielded a significant *P*-value although the interior versus exterior comparison did not. This suggests that unmeasured difference(s) between these broad regions of the country are also associated with CORT_f_ variation.

CORT_f_ was negatively associated with temperatures and precipitation levels, but one-way associations were weak. However, the clustering of the high CORT_f_ birds in the dry north-central region of the country suggests that precipitation is an important ecological variable explaining variation in CORT_f_. Previous studies have found negative associations between CORT_p_ and precipitation levels in white-crowned sparrows (*Zonotrichia leucophrys pugetensis*) in Washington (Wingfield et al. 1983), in dark-eyed juncos (*Junco hyemalis*) wintering across the United States (Rogers et al. 1993), and in Alpine swifts (*Apus melba*) in Switzerland (Bize et al. 2010). House sparrows eat grains primarily but also consume insects (Lowther and Cink 2006). As rainfall is important for plant growth and climate can influence the availability of insect prey (Murphy 1987), the CORT_f_–precipitation associations in the Mexican population could be driven by effects of precipitation on food supply. These sparrows could also be at a risk of dehydration in extremely arid regions. In this case, rainfall could be directly affecting CORT_f_ by increasing the energetic costs of obtaining enough water to maintain homeostasis.

In our study, CORT_f_ showed a negative relationship with temperature and birds with the highest CORT_f_ levels were sampled in a region where annual temperature variation is relatively high. Low ambient temperatures have been associated with higher plasma and fecal CORT levels in multiple species and contexts, including diving petrels (*Pelecanoides urinatrix*) off the coast of South Georgia Island (Smith et al. 1994), Greylag geese in Austria (Frigerio et al. 2004), and nestling blue tits (*Cyanistes caeruleus*) and pied flycatchers (*Ficedula hypoleuca*) in central Spain (Lobato et al. 2008). Additionally, tree swallow (*Tachycineta bicolor*) nestlings that experienced greater temperature variability within nest boxes had higher CORT_f_ levels (Fairhurst et al. 2012), suggesting that CORT_f_ reflects differences in the range of temperatures experienced, in addition to differences in absolute temperatures. These associations likely reflect effects of ambient temperature on energetic requirements or food availability. During the molt period, extreme minimum temperatures at our sampling sites ranged from 8.2°C for June to 1.4°C for November and the lower bound of the thermoneutral zone for house sparrows has been designated as 20–22°C (Hudson and Kimzey 1966).

Interestingly, CORT_f_ was not most strongly related to climate variables during the molt period although CORT_f_ reflects energetic condition during feather growth (Fairhurst 2011). This suggests that conditions experienced during the wintering or breeding stages could have effects that carry over to influence energetic state during molt. For example, cool temperatures during the pre-breeding or breeding stages could cause birds to expend more energy to maintain their body temperature, resulting in reduced fat storage. Unless these negative effects can be countered by increased fat storage during breeding, the individual would be in relatively poor condition during the feather growth period, which could result in higher CORT_f_. Alternately, extreme temperatures or precipitation levels earlier in the year could determine the quantity or quality of food available during the breeding and molt periods.

## Conclusions

We found broad spatial patterns in house sparrow CORT_f_ levels and linked variation in CORT_f_ to climatic variables, indicating that CORT_f_ can enhance our understanding of physiological limitations to avian distributions. Our results suggests that assessing CORT_f_–climate relationships can inform how invasive and non-invasive species will respond to climate change, critical knowledge required to minimize their impacts on native species and the habitats on which they depend. Additionally, this work shows that previously described relationships between climate variables and plasma or fecal CORT can be detected using CORT_f_, adding to a growing body of evidence highlighting the utility of CORT_f_ for assessing how birds cope with environmental challenges.

## Supporting Information

**Figure S1.** Feather CORT values of house sparrows (*Passer domesticus*) sampled in Mexico plotted as contour lines on the reduced space created by two axes produced by ordination of 37 climate variables.

**Figure S2.** Serial dilutions (1:1, 1:2, and 1:4) of four house sparrow feather extracts plotted against a 4-parameter standard curve fitted to nine corticosterone (CORT) standards ranging in concentration from 3.125 to 800 pg/100 µL.

**Table S1.** Results of linear regressions between feather corticosterone (CORT_f_) of house sparrows sampled from 49 sites across Mexico and measures of monthly temperature, precipitation, and deuterium excess (*d*-excess).

**Table S2.** Recovery efficiencies of five methanol extractions used to extract corticosterone from house sparrow feathers (*n* = 448).

**Table S3.** Inter-assay comparisons of ten radioimmunoassays used to measure corticosterone (CORT) levels of house sparrow feather extracts (*n* = 448 samples).

**Appendix S1-S2.** Complete captions of Figures S1, S2 and Tables S1–S3.
