# Supplementary figures and images for: Large-scale spatial variation in feather corticosterone in invasive house sparrows (Passer domesticus) in Mexico is related to climate
Source: Ecol Evol. 2015 Aug 21;5(17):3808–17. doi: 10.1002/ece3.1638 (PMC4567882; doi:10.1002/ece3.1638)

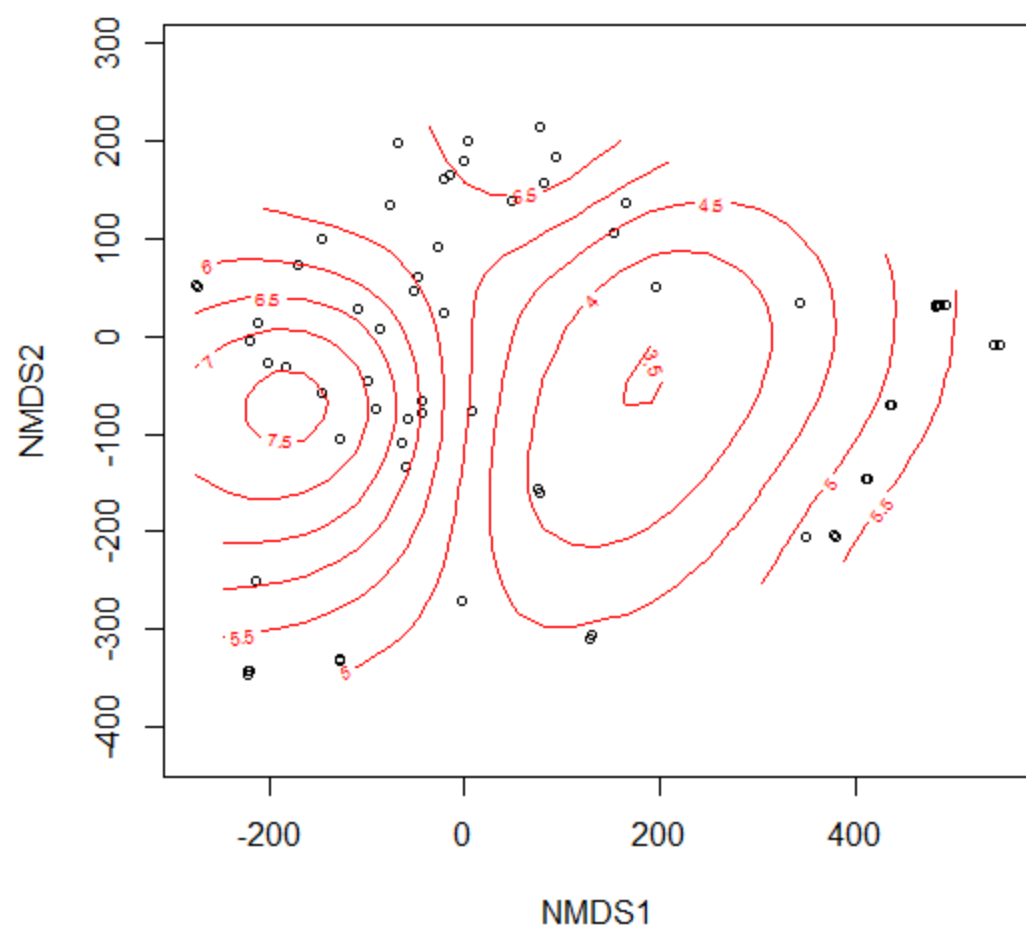

Supplement: Supplementary file 1 — Figure S1. Feather CORT values of house sparrows (Passer domesticus) sampled in Mexico plotted as contour lines on the reduced space created by two axes produced by ordination of 37 climate variables. [file ece30005-3808-sd1.pdf]

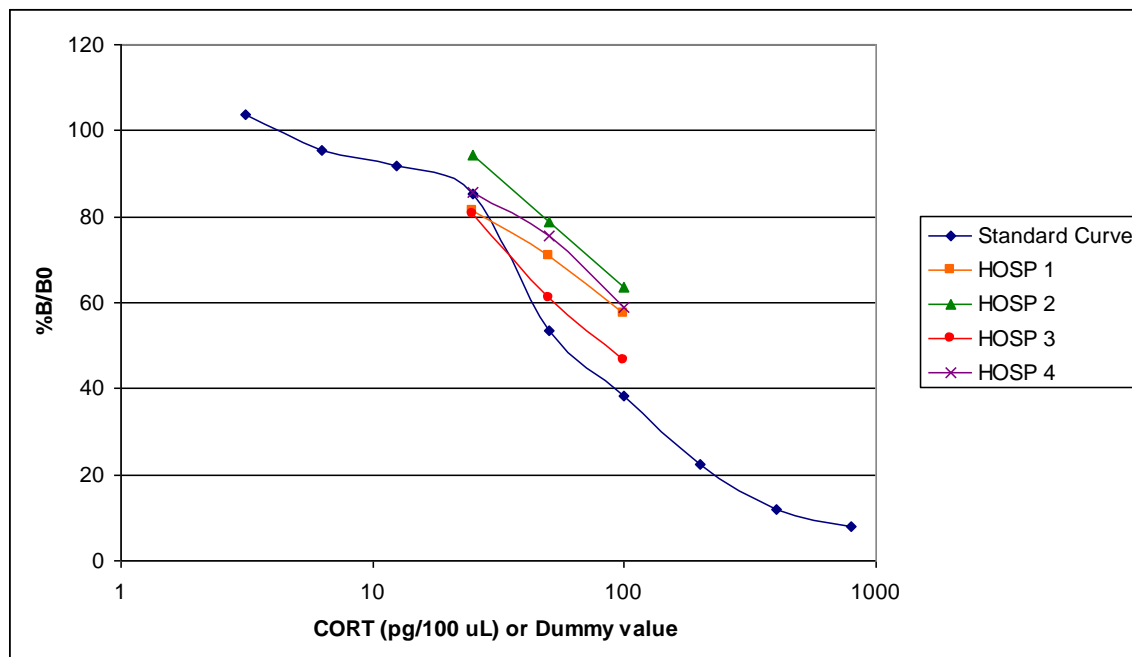

Supplement: Supplementary file 2 — Figure S2. Serial dilutions (1:1, 1:2, and 1:4) of four house sparrow feather extracts plotted against a 4-parameter standard curve fitted to nine corticosterone (CORT) standards ranging in concentration from 3.125 to 800 pg/100 µL. [file ece30005-3808-sd2.pdf]
