# Supplementary material for: Large-scale spatial variation in feather corticosterone in invasive house sparrows (Passer domesticus) in Mexico is related to climate
Source: Ecol Evol. 2015 Aug 21;5(17):3808–17. doi: 10.1002/ece3.1638 (PMC4567882; doi:10.1002/ece3.1638)
Supplement: Supplementary file 3 — Table S1. Results of linear regressions between feather corticosterone (CORTf) of house sparrows sampled from 49 sites across Mexico and measures of monthly temperature, precipitation, and deuterium excess (d-excess). [file ece30005-3808-sd3.docx]

| **Explanatory Variable** | **R squared** | **P-value** | **Explanatory Variable** | **R squared** | **P-value** |
| --- | --- | --- | --- | --- | --- |
| Avg. Jan. precipitation | 0.0125 | 0.0180 | Avg. max. Jan. temp. | 0.0756 | 3.29 x 10^-9^ |
| Avg. Feb. precipitation | 0.0022 | 0.3240 | Avg. max. Feb. temp. | 0.0777 | 1.93 x 10^-9^ |
| Avg. March precipitation | 0.0152 | 0.0089 | Avg. max. March temp. | 0.0633 | 6.74 x 10^-8^ |
| Avg. April precipitation | 0.0033 | 0.2220 | Avg. max. April temp. | 0.0235 | 0.0011 |
| Avg. May precipitation | 0.0063 | 0.0934 | Avg. max. May temp. | 0.0003 | 0.7210 |
| Avg. June precipitation | 0.0172 | 0.0054 | Avg. max. June temp. | 0.0082 | 0.0548 |
| Avg. July precipitation | 0.0073 | 0.0715 | Avg. max. July temp. | 0.0019 | 0.3610 |
| Avg. Aug. precipitation | 0.0153 | 0.0088 | Avg. max. Aug. temp. | 0.0001 | 0.8190 |
| Avg. Sept. precipitation | 0.0089 | 0.0456 | Avg. max. Sept. temp. | 0.0010 | 0.5110 |
| Avg. Oct. precipitation | 0.0214 | 0.0019 | Avg. max. Oct. temp. | 0.0123 | 0.0189 |
| Avg. Nov. precipitation | 0.0236 | 0.0011 | Avg. max. Nov. temp. | 0.0507 | 1.46 x 10^-6^ |
| Avg. Dec. precipitation | 0.0292 | 0.0003 | Avg. max. Dec. temp. | 0.0697 | 1.39 x 10^-8^ |
| Avg. min. Jan. temp. | 0.0528 | 8.83 x 10 ^-7^ | Jan. *d*-excess | 0.0034 | 0.2160 |
| Avg. min. Feb. temp. | 0.0511 | 1.34 x 10 ^-6^ | Feb. *d*-excess | 0.0056 | 0.1150 |
| Avg. min. March temp. | 0.0443 | 7.11 x 10 ^-6^ | March *d*-excess | 0.0262 | 0.0006 |
| Avg. min. April temp. | 0.0167 | 0.0062 | April *d*-excess | 0.0485 | 2.51 x 10^-6^ |
| Avg. min. May temp. | 0.0077 | 0.0641 | May *d*-excess | 0.0085 | 0.0518 |
| Avg. min. June temp. | 0.0021 | 0.3310 | June *d*-excess | 0.0002 | 0.7730 |
| Avg. min. July temp. | 0.0007 | 0.5680 | July *d*-excess | 0.0019 | 0.3620 |
| Avg. min. Aug. temp. | 0.0021 | 0.3340 | Aug. *d*-excess | 0.0010 | 0.0344 |
| Avg. min. Sept. temp. | 0.0102 | 0.0327 | Sept. *d*-excess | 5.211 x 10^-9^ | 0.9990 |
| Avg. min. Oct. temp. | 0.0263 | 0.0006 | Oct. *d*-excess | 0.0139 | 0.0124 |
| Avg. min. Nov. temp. | 0.0417 | 1.33 x 10 ^-5^ | Nov. *d*-excess | 8.051 x 10^-5^ | 0.8500 |
| Avg. min. Dec. temp. | 0.0521 | 1.06 x 10 ^-6^ | Dec. *d*-excess | 0.0246 | 0.0009 |
