# Supplementary material for: Large-scale spatial variation in feather corticosterone in invasive house sparrows (Passer domesticus) in Mexico is related to climate
Source: Ecol Evol. 2015 Aug 21;5(17):3808–17. doi: 10.1002/ece3.1638 (PMC4567882; doi:10.1002/ece3.1638)
Supplement: Supplementary file 4 — Table S2. Recovery efficiencies of five methanol extractions used to extract corticosterone from house sparrow feathers (n = 448). [file ece30005-3808-sd4.docx]

| **Extraction** | **Recovery Efficiency** |
| --- | --- |
| **1** | 96% |
| **2** | 98% |
| **3** | 99% |
| **4** | 94% |
| **5** | 92% |
| **AVG** | 95.8% |
