# Supplementary material for: Large-scale spatial variation in feather corticosterone in invasive house sparrows (Passer domesticus) in Mexico is related to climate
Source: Ecol Evol. 2015 Aug 21;5(17):3808–17. doi: 10.1002/ece3.1638 (PMC4567882; doi:10.1002/ece3.1638)
Supplement: Supplementary file 5 — Table S3. Inter-assay comparisons of ten radioimmunoassays used to measure corticosterone (CORT) levels of house sparrow feather extracts (n = 448 samples). [file ece30005-3808-sd5.docx]

| **Assay** | **Avg. SIS (pg CORT/100uL)** | **%CV (SIS)** | **% Binding** | **% NSB** | **ED20 (pg CORT/100uL)** | **ED50 (pg CORT/100uL)** | **ED80 (pg CORT/100uL)** |
| --- | --- | --- | --- | --- | --- | --- | --- |
| **1** | 67.46 | 6.28 | 28.06 | 4.12 | 178.31 | 45.69 | 11.71 |
| **2** | 62.24 | 7.33 | 26.45 | 5.63 | 156.56 | 40.71 | 8.11 |
| **3** | 69.40 | 4.50 | 27.47 | 4.99 | 175.04 | 43.57 | 10.83 |
| **4** | 63.78 | 6.98 | 28.28 | 4.23 | 155.25 | 40.96 | 10.89 |
| **5** | 61.97 | 8.15 | 28.20 | 4.24 | 159.94 | 41.12 | 9.89 |
| **6** | 70.24 | 5.41 | 28.92 | 3.97 | 173.04 | 39.60 | 8.82 |
| **7** | 65.77 | 10.60 | 29.19 | 5.63 | 144.72 | 42.50 | 10.45 |
| **8** | 65.09 | 5.01 | 28.58 | 4.33 | 169.24 | 41.46 | 10.01 |
| **9** | 61.63 | 6.36 | 28.08 | 3.59 | 159.90 | 40.37 | 10.18 |
| **10** | 65.52 | 5.04 | 28.13 | 4.12 | 187.68 | 41.52 | 9.18 |
|  |  |  |  |  |  |  |  |
| **Avg.** | 65.31 | 6.57 | 28.14 | 4.49 | 165.97 | 41.75 | 10.01 |
| **%CV** | 4.63 | . | 2.71 | 15.52 | 7.77 | 4.24 | 10.68 |
