# Supplementary material for: Large-scale spatial variation in feather corticosterone in invasive house sparrows (Passer domesticus) in Mexico is related to climate
Source: Ecol Evol. 2015 Aug 21;5(17):3808–17. doi: 10.1002/ece3.1638 (PMC4567882; doi:10.1002/ece3.1638)
Supplement: Supplementary file 6 — Appendix S1-S2. Complete captions of Figures S1, S2 and Tables S1–S3. [file ece30005-3808-sd6.doc]

**SUPPORTING INFORMATION**

**Appendix S1** {Additional Tables and Figures}

Table S1: Results of linear regressions between feather corticosterone (CORT_f_) of house sparrows sampled from 49 sites across Mexico and measures of monthly temperature, precipitation, and deuterium excess (*d*-excess).

Figure S1: Feather CORT values of house sparrows (*Passer domesticus*) sampled in Mexico plotted as contour lines on the reduced space created by two axes produced by ordination of 37 climate variables. Ordination was done using non-metric multidimensional scaling with a Euclidean distance metric, and the solution with the lowest stress consisted of two dimensions or axes. The first axis (NMDS1) is positively correlated with precipitation from months during the rainy season, which overlaps with the moult period of house sparrows, and positively correlated with minimum and maximum temperatures from months outside of the moult period, during the dry season. The second axis (NMDS2) is negatively correlated with precipitation during the moult period or rainy season and positively associated with minimum and maximum temperatures during the moult period or rainy season.

**Appendix S2** {Radioimmunoassay Quality Assurance/Quality Control Data}

Table S2: Recovery efficiencies of five methanol extractions used to extract corticosterone from house sparrow feathers (n=448). Recovery efficiencies were calculated by spiking three feather samples and three empty (total count) vials with ~5000 CPM of ^3^H-corticosterone, subjecting the feather samples to the extraction procedure, then comparing the average amount of ^3^H-corticosterone in the extracted samples to the average amount in the total count vials.

Table S3: Inter-assay comparisons of ten radioimmunoassays used to measure corticosterone (CORT) levels of house sparrow feather extracts (n=448 samples). The ‘SIS’ (steroid internal standard) values are measured from a standard of CORT in phosphate-buffered saline which was run in six tubes per assay. Coefficients of variation (CV) were calculated as the standard deviation divided by the mean. The ‘% NSB’ refers to non-specific binding, and the ED20, ED50, and ED80 are the ‘estimated dose’ of CORT at three points on the standard curve, the levels at which 20%, 50%, and 80% of the CORT in the sample are bound.

Figure S2: Serial dilutions (1:1, 1:2, and 1:4) of four house sparrow feather extracts plotted against a 4-parameter standard curve fitted to nine corticosterone (CORT) standards ranging in concentration from 3.125 – 800 pg/100 µL. Dummy values were used for X variables (100 for 1:1, 50 for 1:2, 25 for 1:4) when plotting the diluted feather extracts in lieu of the CORT values produced by analysis of the data, as these CORT values are based on the standard curve. Visual inspection of the dilution curves indicates that they are parallel to the standard curve, and therefore there are unlikely to be substances in the extracts that might compromised the assay (Bortolotti *et al.* 2008; Buchanan and Goldsmith 2004).
